# Advances in Starch-Based Nanocomposites for Functional Food Systems: Harnessing AI and Nuclear Magnetic Resonance Technologies for Tailored Stability and Bioactivity

**DOI:** 10.3390/foods14050773

**Published:** 2025-02-24

**Authors:** Yue Sun, Ziyu Wang, Jian Ye, Yinta Li, Lili Wang, Ruge Cao

**Affiliations:** 1 College of Food Science and Engineering, Tianjin University of Science and Technology, Tianjin 300457, China; 2 Weihai Key Laboratory of Medical Functional Food Processing Technology, Weihai Ocean Vocational College, Weihai 264300, China; 3 Institute of Food Science and Technology, Chinese Academy of Agricultural Sciences, Ministry of Agriculture, Beijing 100193, China

**Keywords:** starch-based nanocomposites (SNCs), artificial intelligence (AI), nuclear magnetic resonance (NMR), predictive modeling, food matrix

## Abstract

Starch-based nanocomposites (SNCs) are at the forefront of innovations in food science, offering unparalleled opportunities for enhancing the stability, bioactivity, and overall functionality of food systems. This review delves into the potential of SNCs to address contemporary challenges in food formulation, focusing on the synergistic effects of their components. By integrating cutting-edge technologies, such as artificial intelligence (AI) and nuclear magnetic resonance (NMR), we explore new avenues for enhancing the precision, predictability, and functionality of SNCs. AI is applied to optimize the design of SNCs, leveraging predictive modeling to fine-tune material properties and streamline production processes. The role of NMR is also critically examined, with particular emphasis on its capacity to provide high-resolution structural insights, monitor stability over time, and elucidate molecular interactions within food matrices. Through detailed examples, the review highlights the impact of NMR in unraveling the complex behaviors of bioactive compounds encapsulated in SNCs. Additionally, we discuss the integration of functional assays and AI-driven analytics in assessing the bioactivity and sensory properties of these systems, providing a robust framework for the rational design of advanced food products. The synergy between AI, NMR, and SNCs opens new pathways for developing tailored, high-performance food formulations that address both health and consumer preferences.

## 1. Introduction

The food industry is now increasingly tapping into the potential of nanocomposites, particularly in food packaging, ingredient delivery, and food analysis. Among these, starch-based nanocomposites (SNCs) have garnered significant attention due to the natural abundance and biocompatibility of starch, combined with the nanoscale properties that enhance its functionality in food systems [1]. SNCs stand out in the food industry due to their small particle size, high surface-to-volume ratio, and favorable properties such as excellent biocompatibility, biodegradability, and tunable surface chemistry, which are crucial for both functional ingredient delivery and food preservation [2].

At the nanometer scale, starch particles demonstrate significant changes in their surface properties, including an increase in surface area and surface energy. These enhancements in surface properties significantly improve their adsorption capacity and rate, facilitating better loading of bioactive compounds and more efficient controlled release mechanisms. The increased surface-to-volume ratio also improves the efficacy of starch nanoparticles in delivering nutrients, enzymes, and bioactive substances to specific sites within the gastrointestinal tract, thereby enhancing the bioavailability of small molecules [3]. These properties are particularly valuable for developing functional foods and food additives that provide controlled release, targeted delivery, and prolonged action of nutrients or therapeutic agents [4].

Despite the growing promise of SNCs, there remain significant challenges in fully realizing their potential, especially in optimizing their stability and bioactivity in complex food matrices [5]. Traditional characterization techniques, such as X-ray diffraction, transmission electron microscopy, and infrared spectroscopy, have been widely used to study nanocomposites. However, these methods often lack the depth needed to analyze the dynamic, multi-phase interactions that occur in food systems. Recent advances in nuclear magnetic resonance (NMR) and artificial intelligence (AI) are paving the way for a more detailed and predictive approach to nanocomposite analysis [6]. NMR, with its ability to probe the chemical composition and spatial structure of materials, provides a non-destructive and highly detailed examination of complex systems, such as food matrices. It can track molecular interactions, monitor structural changes, and offer insights into the stability and behavior of nanomaterials under various conditions [7]. This makes NMR a powerful tool for advancing food science, where understanding the interplay between ingredients at the molecular level is critical for developing novel food products.

AI, on the other hand, is enhancing the efficiency and precision of nanomaterial development. By integrating AI with experimental data, researchers can predict material properties, optimize formulation processes, and even design new materials with tailored functionalities [8]. In the context of SNCs, AI is particularly promising for optimizing material design, predicting stability over time, and improving the overall bioactivity and sensory characteristics of nanocomposites in food systems [9]. The combination of AI and NMR provides an unprecedented capability to model, test, and refine nanocomposites in a way that accelerates the development of novel food ingredients.

This review explores the recent innovations in the preparation and application of SNCs, with a particular focus on the integration of emerging technologies such as AI and NMR. By examining these technological advances, the review aims to provide a comprehensive overview of how these tools can be used to enhance the stability, bioactivity, and overall functionality of SNCs in food applications. Furthermore, this review discusses the potential future research directions, emphasizing how these advancements could lead to the development of high-performance, nutritionally enriched food systems, and functional food ingredients. The critical analysis of the integration of AI and NMR technologies into the study of SNCs marks this review as an essential resource for researchers looking to advance the field of food nanotechnology and its applications.

## 2. Innovative Fabrication Techniques for SNCs

**Figure 1:**
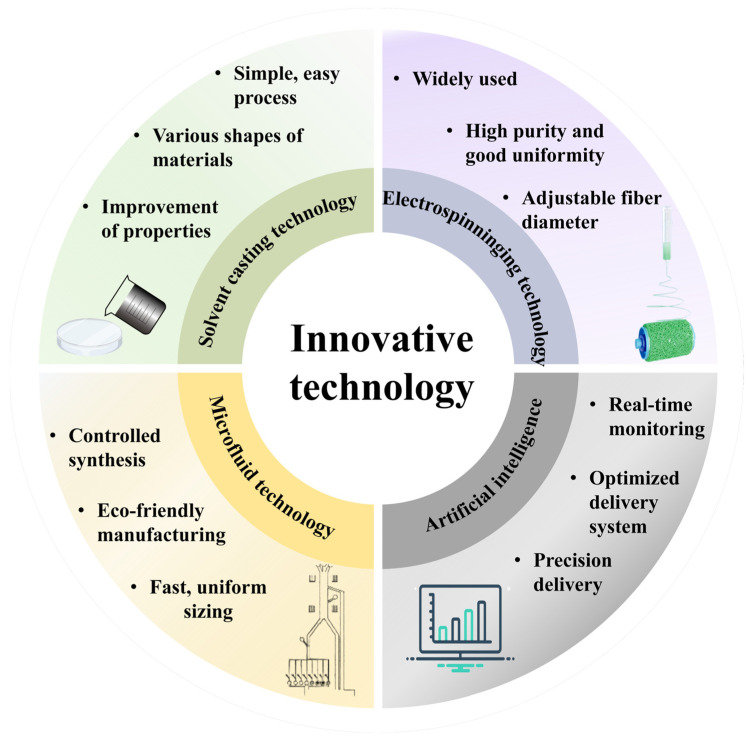
Innovative fabrication techniques for starch-based nanocomposites.

The development of SNCs has witnessed remarkable advances, driven by innovative fabrication techniques. These advances hold immense potential to redefine the role of starch in food systems, offering new solutions for stability, bioactivity, and functionality. The emergence of advanced fabrication methods, particularly microfluidic and electrospinning technologies, offers unprecedented control over the properties of SNCs, allowing for precise tailoring of their characteristics. However, this progress also presents substantial challenges in scaling up production, ensuring reproducibility, and achieving consistency in quality. Despite these hurdles, recent developments underscore the transformative potential of these techniques, which are poised to significantly impact food applications ranging from ingredient delivery to food packaging. Figure 1 illustrates innovative fabrication techniques for SNCs, emphasizing the advantages of each method, including the simplicity of solvent casting technology for preparing complex nanocomposite shapes. Solvent casting technology, for instance, is particularly beneficial due to its low equipment requirements and ability to produce nanocomposites with complex shapes. Electrospinning, on the other hand, yields fibers with high purity, uniformity, and a wide range of applications. Microfluidic technology enables precise control over particle size, size distribution, and morphology of nanocomposites. Lastly, AI enhances material development by predicting properties, optimizing composition and structural design, and providing real-time monitoring.

### 2.1. Comparison Between Traditional and Innovative Methods

The fabrication of SNCs has long been a cornerstone of nanomaterials research, with traditional methods such as acid hydrolysis, enzymatic hydrolysis, emulsion cross-linking, precipitation, and recrystallization playing essential roles in modifying starch granules to generate nanoparticles. While these techniques have proven effective in producing SNCs with varying sizes and properties, they are not without limitations. These traditional methods typically struggle with precision, scalability, and consistency in particle size distribution, which are crucial for applications in food systems where uniformity and stability are paramount.

For example, acid hydrolysis, a commonly used method, effectively reduces starch to smaller particles but often leads to poor control over the uniformity of particle size. This results in a wide size distribution, which compromises the reproducibility and stability of the SNCs. Additionally, the harsh chemical conditions required in acid hydrolysis can degrade the structural integrity of the starch, limiting the functional performance of the resulting SNCs [10]. Enzymatic hydrolysis, while milder, also faces challenges in terms of reaction time and enzyme specificity, which can further hinder the precision required for high-quality nanoparticle synthesis [11]. Furthermore, methods such as precipitation and recrystallization often fail to achieve the desired particle size and morphology, particularly when scaling up for industrial applications [12]. These challenges underscore the need for more refined, efficient, and scalable techniques to produce SNCs with tailored properties for specific applications, especially in complex food matrices.

In response to these limitations, emerging fabrication techniques, such as microfluidic technology and electrospinning, have gained significant attention due to their ability to offer higher precision in controlling particle size, morphology, and distribution. These advanced methods address the shortcomings of traditional approaches by providing more consistent, scalable, and reproducible means of fabricating SNCs, making them ideal for sophisticated food applications, including controlled release systems and sustainable packaging solutions [13]. Table 1 compares traditional and innovative fabrication methods for SNCs, highlighting their respective advantages and limitations.

Microfluidics, which involves the manipulation of fluids at the microscale on compact chips, has fundamentally changed the landscape of nanomaterial fabrication. This technique enabled precise control over particle synthesis. In the context of SNCs, microfluidic platforms provide unparalleled accuracy in controlling the size, distribution, and morphology of nanoparticles. It allows for the synthesis of monodisperse nanoparticles with narrow size distributions, critical for achieving consistency in food applications. The use of microfluidic devices also allows for multi-step processes that can produce complex nanocomposite structures, such as core–shell nanoparticles or hybrid hydrogels, which can be used in food packaging, ingredient encapsulation, and delivery systems [11]. Research has demonstrated the superiority of microfluidic technology in controlling nanoparticle characteristics. For instance, Chen et al. [14] prepared high-performance starch-based hydrogels via microfluidic technology, achieving significant improvements in mechanical properties and conductivity, which are important attributes for food packaging materials that need to withstand environmental stress. Furthermore, Yang et al. [15] highlighted how microfluidic platforms enable the uniform dispersion of starch nanoparticles, resulting in films with improved mechanical strength and transparency, ideal for food packaging that requires both durability and visual appeal.

Another innovative fabrication technique is electrospinning, which uses electrostatic forces to transform polymer solutions into micro- and nanoscale fibers. In the context of SNCs, electrospinning offers a promising route for creating starch-based nanofibers with unique structural properties that enhance their functionality in food systems [16]. One of the key advantages of electrospinning is its non-thermal nature, which preserves the integrity of sensitive bioactive compounds that might degrade under traditional thermal processing methods, such as spray drying. This is particularly valuable in the food industry, where the controlled release of bioactive agents (such as antioxidants, preservatives, or probiotics) is critical for improving food safety and extending shelf life. Zhang et al. [17] demonstrated how electrospun starch fibers can be used in active food packaging, highlighting their ability to release bioactive compounds in a controlled manner, thus enhancing food preservation while reducing packaging waste. Huang et al. [18] further emphasized that electrospun starch nanofibers exhibit high stability and excellent thermal decomposition characteristics, making them ideal for high-performance food packaging materials. Moreover, Lv et al. [19] showed that starch-based nanofiber mats, prepared by electrospinning and cross-linking with catechins, can achieve high encapsulation efficiency and improved antibacterial properties, offering a novel approach to antimicrobial food packaging.

**Table 1:** Comparison of traditional and innovative fabrication methods for SNCs.

	Fabrication Technique	Advantages	Limitations	Refs
Traditional technology	Self-assembly method	Structural controllability, convenience and environmental friendliness, wide applicability	Complex influencing factors, template dependence, limitations of physical field regulation	[20]
	Cross-linking method	Good biocompatibility, biodegradability	High equipment requirements, applicability restriction, non-uniform particle size, may introduce toxic substances	[21]
	Precipitation method	Easy to operate, short reaction time, low cost, wide applicability	Low purity, large particle radius, agglomeration, organic solvent residue	[22,23]
	Mechanical method	Wide applicability, scale of production, efficient and convenient, small and uniform size	High equipment requirements, high energy consumption, easily mixed with impurities	[24]
Innovative technology	Microfluidic technology	Controllable particle size and morphology, short preparation time, high yield, precise control of reaction conditions, less reagent needed	High equipment cost and maintenance cost, challenges of large-scale production, technical challenge	[25]
	Electrostatic spinning technology	Adjustable fineness, high production efficiency, diversification of fiber material, easy to control, wide applicability	Industrialization application challenge, high cost, complex influencing factors	[26]
	Solvent casting technology	Simple operation, high porosity, controllable pore structure, wide applicability	Solvent selection restriction, possible solvent residue, environmental and health issues, high equipment cost	[27]

### 2.2. Application of AI in SNCs Design and Optimization

#### 2.2.1. Model Prediction and Rapid Analysis

AI-driven predictive modeling significantly accelerates the design and optimization of SNCs for food applications, improving thermal stability, mechanical strength, and bioactive release rates before synthesis, and minimizing trial-and-error testing [28,29,30]. For example, Dubrovsky et al. [31] used Graph Neural Networks (GNNs) and Generative Adversarial Networks (GANs) to predict the size and morphology of nanomaterials, which directly impact their food system compatibility. These AI models help predict and optimize SNCs to meet food industry standards in terms of both performance and cost-effectiveness. Additionally, Vargo et al. [32] explored how Machine Learning (ML) models could predict the self-assembly behavior of nanocomposites, optimizing their mechanical properties and interactions in complex food systems, offering a more efficient means of formulation development.

#### 2.2.2. Industrial Production

Scaling up the production of SNCs to meet food industry demands requires precise control over production conditions, which is where AI proves invaluable. For instance, Mottafegh et al. [33] developed an autonomous multi-step platform that uses ML to optimize flow rate, temperature, and pH during production, ensuring consistent quality and cost-effective scaling for food-grade SNCs. This innovation directly benefits the food industry by reducing production time and costs, while enhancing material consistency for large-scale applications. Similarly, Du et al. [34] highlighted how data-driven modeling enables real-time monitoring and optimization in industrial production, improving the efficiency and scalability of SNCs manufacturing for food use. These case studies highlight how AI is driving efficiency and cost savings in the production of SNCs, ultimately improving the scalability and economic viability of SNCs applications in the food industry.

## 3. Application of NMR in SNCs

Nuclear magnetic resonance (NMR) refers to the phenomenon in which nuclei with nonzero spin quantum numbers, such as ^1^H, ^13^C, and ^19^F, interact with an alternating magnetic field while under the influence of a constant magnetic field, leading to energy exchange [35,36]. In SNCs research, high-field NMR provides atomic- and molecular-level structural information, enabling researchers to elucidate intricate molecular interactions and material dynamics [37,38].

### 3.1. Structural Analysis of SNCs Using NMR

Recent advancements in high-field NMR have enhanced its ability to analyze the molecular structure and interactions within SNCs. Ma et al. [39] used ^13^C CP/MAS two-dimensional solid-state NMR (ssNMR) to examine molecular interactions between amylose and polyphenols. ^13^C CP/MAS and ^1^H-^13^C heteronuclear correlation (HETCOR) pulse sequences were optimized with a contact time of 5 ms and a repetition time of 30 s for detecting hydrogen bonds. This study provided direct evidence of stable amylose–polyphenol complexes, crucial for designing polyphenol delivery systems based on SNCs. Similarly, Wang et al. [40] employed ^1^H NMR, ^13^C CP/MAS, and ^1^H-^13^C HETCOR to investigate the interaction between digestive enzymes and banana-resistant starch nanoparticles, revealing the stability and bioactive release behavior of SNCs during digestion.

Moreover, Dong et al. [41] used ^1^H and ^13^C NMR, Correlation Spectroscopy (COSY), Heteronuclear Single Quantum Coherence (HSQC), and Heteronuclear Multiple Bond Correlation Spectroscopy (HMBC) to study structural changes in corn, pea, and cassava starch, especially the rearrangement of α-1,4 and α-1,6 glycosidic bonds. These NMR techniques successfully identified the key molecular rearrangements in starch and linked them to changes in the mechanical properties of starch-based films. These examples demonstrate the power of NMR to provide deep insights into molecular interactions and material dynamics, essential for optimizing SNCs in food applications.

### 3.2. Non-Destructive Analysis of SNCs Using NMR

One of the greatest advantages of NMR is its non-destructive nature, which allows for repeated measurements and long-term monitoring of dynamic processes without compromising the integrity of the sample [35]. This makes NMR especially valuable in the food industry, where ensuring the stability and consistency of ingredients is critical. Wang et al. [42] used COSY and HSQC to investigate hydrogen bonding and π-π stacking interactions between curcumin and SNCs. The study revealed how these molecular interactions contribute to the stability and bioavailability of curcumin, which is crucial for designing effective delivery systems.

Additionally, Yan et al. [43] employed COSY, HSQC, and ^13^C CP/MAS NMR techniques to study structural changes in starch nanoparticles after debranching treatment, providing insights into how modifications affect the crystallinity and properties of the material. Shin et al. [44] employed ssNMR to investigate how fatty acid chain length influences the structure of SNCs. ^1^H and ^13^C NMR spectra confirmed the successful embedding of fatty acids within the starch matrix: COSY revealed proton coupling relationships, identifying how fatty acid chain length affects composite structure; HSQC analyzed proton–carbon interactions; ^13^C CP/MAS NMR evaluated the crystallinity of SNCs and the effect of fatty acid incorporation on the crystal structure of composites. Similarly, Meng et al. [45] used NMR to monitor OSA-modified SNCs. ^13^C CP/MAS NMR identified chemical changes, while the C_1_ and C_4_ signal regions were analyzed to confirm alterations in crystal structure and resistant starch content. Real-time tracking of proton and carbon shifts highlighted the stability of SNCs during modification, demonstrating the non-destructive capabilities of NMR.

In the food industry, NMR spectroscopy has been applied for real-time molecular composition analysis, ensuring quality control in processed foods such as fruit, meat, wine, and spices [46]. NMR was also used to monitor bioactive ingredient stability in beverages, preventing degradation and preserving nutritional quality [47]. These applications underscore the role of NMR in advancing both SNCs research and food industry quality assurance.

## 4. Mechanistic Insights into Bioactivity of SNCs

The bioactivity of SNCs is a critical determinant of their functional properties, stability, and reliability in food systems [48]. Understanding the molecular mechanisms underlying the interactions of SNCs and the factors affecting their bioactivity provides essential theoretical and practical insights for optimizing their design and applications. This section explores key interaction mechanisms within SNCs systems, factors influencing their bioactivity, and the transformative role of AI in advancing the stability and application of SNCs in food science.

**Figure 2:**
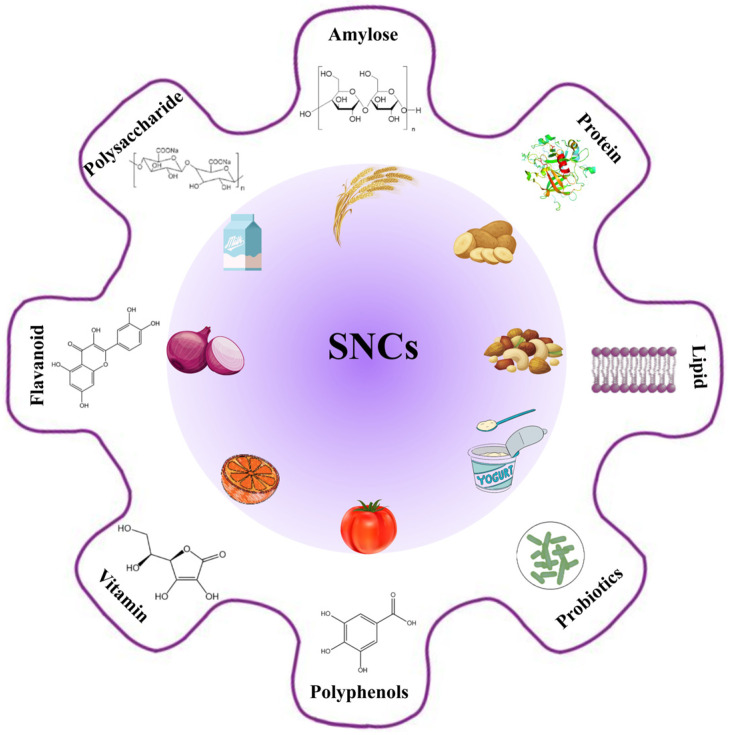
Common components and their dietary sources in starch-based nanocomposites.

To fully understand the bioactivity of SNCs, it is crucial to examine the key components commonly present in these nanocomposites, as they significantly affect their functionality and interactions within food systems. SNCs typically incorporate bioactive substances such as proteins, polysaccharides, polyphenols, and vitamins, each contributing unique structural and functional properties. Proteins enhance the mechanical strength and stability of SNCs [49], while polysaccharides improve water retention and biocompatibility [50]. Polyphenols, known for their antioxidant and antimicrobial properties, broaden the applications of SNCs in food preservation and health-promoting formulations [51]. Figure 2 highlights common components in SNCs, including key bioactive substances and their dietary sources.

### 4.1. Molecular Mechanisms of Interaction in SNCs Systems

Hydrogen bonding between starch and polysaccharides plays a critical role in enhancing the thermal stability and biocompatibility of SNCs. This interaction has been demonstrated in multiple studies. Su et al. [52] found that hydrogen bonds between starch and polysaccharides, such as cellulose and guar gum, significantly improved the thermal stability of the composites, making them suitable for food packaging applications. These interactions help form stronger composite structures, stabilizing encapsulated bioactive molecules. Hu [53] and Chen [54] further corroborated these findings, emphasizing the essential role of hydrogen bonds in stabilizing composite structures.

In addition, electrostatic interactions play a significant role in SNCs, particularly in the encapsulation and controlled release of functional molecules. Zhang et al. [55] explored the interaction between starch and anionic polysaccharides such as alginate and chitosan. They found that the electrostatic attraction between the positively charged starch molecules and negatively charged polysaccharides resulted in the formation of stable composite structures. This interaction enabled the effective encapsulation of polyphenolic compounds and their controlled release, showcasing the potential of SNCs in targeted delivery systems. Zhou [56] and Xu [57] also demonstrated the crucial role of electrostatic interactions in the stability and functionality of SNCs.

Molecular rearrangement also plays a vital role in the resistance of SNCs to enzymatic hydrolysis. Qiu et al. [58] studied ternary composites composed of starch, lipids, and chlorogenic acid. Their findings showed that hydrophobic interactions were the main driving force for the formation of SNCs, promoting molecular rearrangement and enhancing structural stability. This rearrangement improved the encapsulation efficiency, stability, and digestibility of chlorogenic acid. He [59] and Ma [60] made similar observations, highlighting how molecular interactions enhance the bioavailability and efficacy of bioactive ingredients in food systems.

Recent advancements have also highlighted the role of cross-linking agents in enhancing the stability and functionality of SNCs. For instance, Zhang et al. [61] investigated the use of tannic acid as a cross-linking agent in starch biopolymer-based food packaging films. Their study showed that tannic acid induced additional intermolecular interactions between starch molecules, resulting in more robust and thermally stable structures. This cross-linking significantly enhanced the encapsulation of antioxidants, suggesting its potential in functional foods designed for therapeutic applications. Xie et al. [62] developed a novel starch nanofiber membrane through self-assembly using acylated tannic acid at the interface. This modification endowed the membrane with antibacterial, antioxidant, and UV-blocking properties. The use of cross-linking agents like tannic acid can significantly enhance the stability, functionality, and biological activity of SNCs, promoting their potential for food packaging and functional food applications.

**Figure 3:**
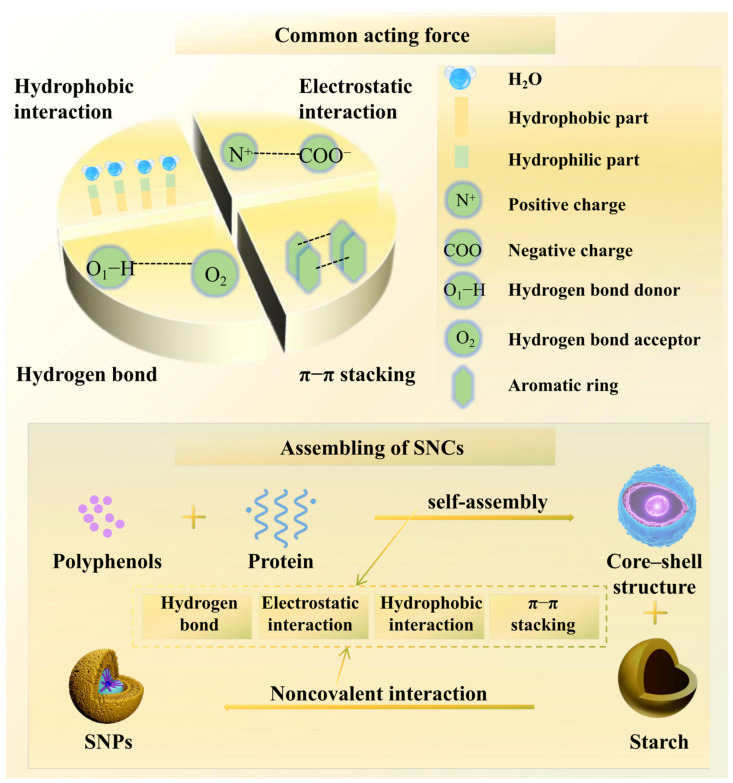
Basic interactions between molecules in SNCs.

These studies collectively demonstrate the complex molecular interactions that govern the formation and stability of SNCs. Figure 3 visualizes molecular interactions critical to the formation of SNCs, providing insight into their structural assembly and stability, which are key to optimizing functionality in diverse applications. Understanding these mechanisms is essential for optimizing the preparation processes of SNCs and enhancing their functional properties for food applications. While molecular interactions such as hydrogen bonding, electrostatic forces, and cross-linking define the stability and functionality of SNCs, their bioactivity is further influenced by intrinsic and extrinsic factors.

### 4.2. Factors Influencing the Bioactivity of SNCs

**Figure 4:**
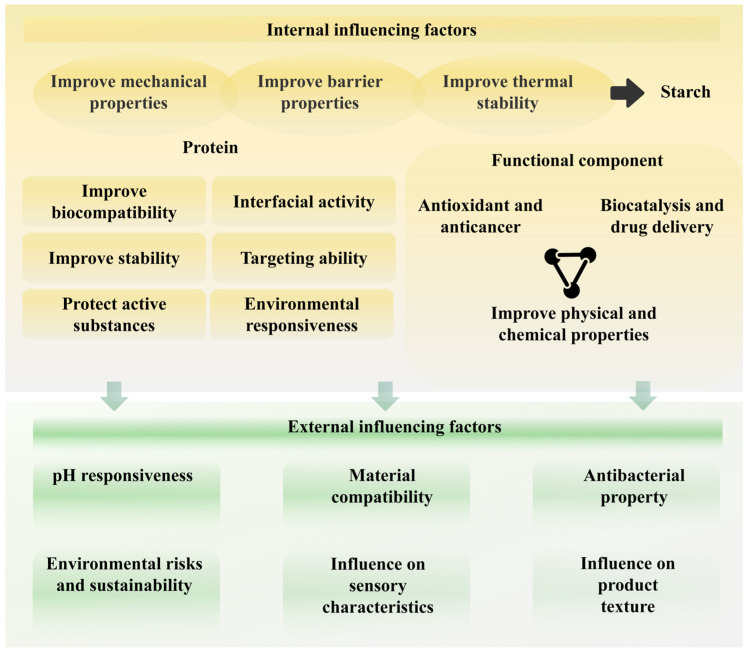
Key factors influencing bioactivity and stability of starch-based nanocomposites.

SNCs have gained considerable attention in recent years due to their bioactivity, which is influenced by both intrinsic and extrinsic factors. The bioactivity of SNCs, particularly in terms of their interaction with biological systems, is critical for their applications in various fields, such as drug delivery, food packaging, and tissue engineering. These factors can impact the stability of materials, biodegradability, and biocompatibility, all of which are essential for the successful deployment of SNCs in practical applications. Figure 4 demonstrates how both intrinsic and extrinsic factors influence the bioactivity and stability of SNCs, offering a comprehensive framework for guiding future research efforts.

#### 4.2.1. Intrinsic Factors

##### Plant Source

The botanical source of starch is a key determinant of the structural and functional properties of SNCs [63]. Different starches, such as those from corn, potato, tapioca, and rice, possess distinct amylose-to-amylopectin ratios, which significantly influence the behavior of SNCs. High-amylose starches typically form more rigid, crystalline nanoparticle structures, enhancing stability but reducing solubility and bioactivity [64]. In contrast, starches with higher amylopectin content form more flexible, amorphous nanoparticles, which tend to have better interaction with biological systems and higher drug encapsulation efficiency [65]. For instance, Nambiar et al. [66] mentioned in their review that the structure and properties of SNCs can be optimized by adjusting the amylose-to-amylopectin ratio.

##### Particle Morphology and Size

The size and morphology of SNCs are critical factors influencing their bioactivity. Smaller, spherical, or rod-like particles tend to have a larger surface area, facilitating better interaction with biological cells and improving their ability to cross cellular barriers [67]. Apostolidis et al. [68] prepared spherical resistant starch nanoparticles through physical methods like gelatinization, precipitation, and ultrasonic treatment. Their results showed that the addition of spherical nanoparticles reduced surface tension and improved the colloidal stability of emulsions. Similarly, Yang et al. [69] investigated the interaction between SNCs and soy protein isolate (SPI), showing that smaller SNCs adsorb more effectively onto protein surfaces, forming a stable protein crown and enhancing thermal and mechanical stability.

##### Composition Selection

The inclusion of proteins or bioactive molecules in SNCs can significantly enhance their bioactivity. For example, incorporating albumin or casein improves the stability and bioavailability of SNCs by preventing aggregation. Furthermore, bioactive molecules such as astaxanthin, quercetin, and xanthophyll can be encapsulated within SNCs to enhance their antioxidant capacity and bioavailability. Research by Lv et al. [70] demonstrated that astaxanthin-loaded SNCs exhibited superior antioxidant activity compared to free astaxanthin, potentially benefiting the treatment of oxidative stress-related diseases. These modifications improve both the bioactivity and controlled release of bioactive compounds in SNCs.

#### 4.2.2. Extrinsic Factors

##### pH Responsiveness

The ability of SNCs to respond to pH changes is particularly valuable for applications such as drug delivery, where different body regions have varying pH levels (e.g., acidic stomach vs. neutral intestine). Zhou et al. [71] developed pH-responsive cationic SNCs that remain stable in neutral and alkaline conditions but swell and break in acidic environments, making them suitable for targeting the stomach and upper gastrointestinal tract. Similarly, Bora et al. [72] developed a starch/gelatin/itaconic acid pH-sensitive drug carrier via chemical cross-linking. Their system demonstrated sustained drug release, improving the therapeutic efficacy of the drug under controlled pH conditions.

##### Environmental Impact and Sustainability

As demand for sustainable, eco-friendly materials grows, the environmental impact of SNCs has become an important consideration. Starch, being a biodegradable and renewable resource, makes SNCs an attractive alternative to synthetic nanomaterials, which may pose environmental risks. Moreover, biodegradable SNCs break down into non-toxic by-products, minimizing long-term environmental harm. Yao et al. [73] demonstrated that SNCs degrade rapidly in soil and water, reducing environmental accumulation. This biodegradability also contributes to biocompatibility, as SNCs are less likely to accumulate in the body and cause adverse effects. Their sustainable nature also aligns with the growing demand for eco-friendly materials in industries like packaging and food processing.

##### Biocompatibility and Toxicity

Biocompatibility is crucial when designing SNCs for biomedical applications to ensure they do not provoke an immune response or cause toxicity. SNCs derived from natural starch sources, such as corn or potato, are known for their excellent biocompatibility, which is essential for drug delivery and tissue engineering [74]. For instance, Mei et al. [75] demonstrated that the delivery systems of SNCs are highly compatible and biodegradable, effectively loading anti-cancer drugs like paclitaxel and adriamycin, enhancing drug efficacy while reducing side effects through targeted delivery. Similarly, Dhayal et al. [76] found that SNCs, modified via esterification and etherification, exhibited enhanced biocompatibility and cellular uptake, reducing immunogenicity and improving therapeutic potential. The ability to tailor the surface properties of SNCs further enhances their compatibility with biological systems, minimizing the risk of adverse reactions.

### 4.3. AI-Driven Innovations in SNCs Stability and Food Applications

Traditional experimental methods for evaluating SNCs stability can be slow and resource-intensive. AI, especially ML and deep learning (DL) models, are now used to accelerate stability predictions, optimize formulations, and simulate molecular interactions to improve their bioactivity in food systems. Griesemer et al. [77] demonstrated the efficacy of ML in predicting thermodynamic properties, thereby streamlining the stability assessment of nanomaterials. AI-powered molecular dynamics simulations offer a robust framework for investigating the interactions of SNCs at the molecular level. For instance, Li et al. [78] utilized these simulations to elucidate the stability mechanisms of complexes formed between V_7_ short amylopectin and resveratrol, identifying hydrogen bonding as a critical stabilizing force. Similarly, Zhao et al. [79] employed AI-enhanced simulations to confirm that amylose–piperine complexes are stabilized by hydrogen bonds, illustrating how AI can elucidate intricate molecular behaviors. The capability of AI to simulate and predict SNCs behavior extends beyond stability to applications in functional foods, offering new insights and opportunities for innovation in this field.

## 5. Potential Applications of SNCs in Advanced Functional Foods

### 5.1. Enhancing Nutritional Value and Functional Properties of SNCs

The growing demand for natural, nutritious, and sustainable food options [80] has accelerated the development of SNCs as a transformative tool in functional food innovation. SNCs systems offer unique advantages, including the ability to encapsulate bioactive components, protect sensitive nutrients, and enable controlled release and targeted delivery [81]. These features not only enhance the nutritional value of food products but also align with consumer preferences for health-oriented solutions. Furthermore, the multifunctionality of SNCs extends to applications in food packaging and as fat substitutes, demonstrating their potential to reshape the landscape of modern functional foods. Table 2 showcases recent advancements of SNCs in food applications, providing a snapshot of key developments in the field.

#### 5.1.1. Food Packaging

The unique surface properties of SNCs, characterized by abundant hydroxyl groups and a robust hydrogen bond network, make them an excellent material for developing high-performance food packaging films. These films exhibit enhanced mechanical strength, moisture resistance, and oxygen barrier properties, providing innovative solutions for extending food shelf life and ensuring safety.

Recent advancements have utilized SNCs to create environmentally friendly and biodegradable packaging materials. Li et al. [98] developed a novel carboxymethyl cellulose/starch/anthocyanin/zinc oxide nanoparticle active film, which exhibits excellent mechanical properties and functional characteristics. Additionally, Othman et al. [99] explored the progress of incorporating chitosan nanoparticles into starch-based biopolymer films, which not only foster the development of sustainable materials but also reduce environmental impact and enhance the industrial innovation of nanocomposite technology. In another application, Sganzerla et al. [100] developed bioactive packaging materials based on starch, citric acid pectin, and *Acca sellowiana* waste by-products, applied to apple postharvest preservation. The results showed that this bioactive packaging significantly slowed down the respiration and ethylene release of apples, effectively extending their shelf life. The packaging materials also demonstrated good biodegradability, meeting the growing demand for sustainable products. These SNC-based materials not only enhance food preservation and reduce reliance on synthetic additives, but also align with consumer preferences for clean-label products and sustainability. Currently, SNC-based materials are used in fruit, meat, and bread preservation [101,102].

#### 5.1.2. Fat Substitutes

SNCs present a promising solution for developing low-fat or zero-fat alternatives, addressing the rising demand for healthier food options without compromising on taste or texture. By manipulating the particle size and structural attributes of SNCs, their ability to adsorb water and oil can be precisely tailored to mimic the sensory properties of traditional fats, making them ideal substitutes across various food categories.

Miao et al. [103] developed an octenyl succinic anhydride starch/chitosan oil gel as a fat replacement, demonstrating that the adsorption properties of this complex effectively inhibited lipid oxidation and hydrolysis, highlighting its potential as a solid fat substitute in the food industry. Xu et al. [104] explored the functionality and applications of SNCs and other emulsion gels in a fat substitution strategy for dairy products. Several studies have shown that emulsion gels are excellent fat substitutes in dairy products and represent a promising trend for the future of the dairy industry. Lin et al. [105] summarized the effectiveness of SNCs, such as gels, in replacing fats, noting that SNC-based fat substitutes can replicate the rheological properties and texture of fat, successfully reducing fat content without compromising the sensory qualities of food. Additionally, these findings highlight the versatility of SNCs in meeting health and functional demands in dairy, baked goods, and meat substitutes. As fat substitutes, SNCs extend their benefits beyond their physical properties to their nutritional benefits. The integration of SNC-derived fat replacers in food products to effectively reduce total fat and caloric content has now been successfully applied across a wide range of products [106]. With significantly lower caloric content than traditional fats, SNCs support the development of calorie-reduced and nutrient-enriched food products, enabling manufacturers to align with consumer preferences for balanced and health-conscious diets.

### 5.2. AI Applications in SNCs Design and Consumer-Driven Innovations

The AI and food technology field is redefining how functional foods are designed, marketed, and consumed. By leveraging AI-driven insights, manufacturers can seamlessly connect the functional properties of SNCs with evolving consumer demands and market trends. This integration enables real-time adaptability, precise product personalization, and the creation of health-oriented solutions tailored to modern lifestyles. AI not only facilitates customized product development but also enhances sustainability and consumer satisfaction by aligning innovations with market needs.

#### 5.2.1. Leveraging Data Mining for Consumer-Centric Innovation

Data mining technologies serve as a foundation for extracting actionable insights from extensive datasets, enabling a granular understanding of consumer behaviors and preferences. By analyzing patterns such as purchasing habits, health concerns, and emerging dietary trends, food manufacturers can develop targeted strategies for personalized product offerings.

For example, Chen et al. [107] applied data mining alongside the Theory of Planned Behavior to explore consumer attitudes toward plant-based meat, providing a framework for understanding the potential of starch-based compounds as fat substitutes in functional food applications. Similarly, Boateng et al. [108] highlighted the role of advanced data processing technologies in intelligent sensing systems, demonstrating how real-time consumer data can guide the development of SNC-based solutions for health-focused markets. These insights also facilitate the integration of SNCs into sustainable food systems, ensuring that innovations align with both consumer expectations and environmental priorities.

#### 5.2.2. Transformative Potential of ML in SNCs Optimization

ML algorithms elevate the capabilities of AI by enabling predictive modeling and optimization of both consumer behaviors and product functionalities. In the context of SNCs, ML provides the tools to refine nanoparticle properties for enhanced performance while simultaneously predicting market dynamics to ensure product success.

Li et al. [109] demonstrated how AI optimization can improve the design of biocompatible hydrogels for precise drug delivery, offering a parallel for SNCs in nutrient delivery systems. Similarly, Boateng et al. [110] showcased the application of convolutional neural network algorithms in developing flexible strain sensors, achieving a remarkable 96.3% accuracy in aerial handwriting recognition. These studies highlight the adaptability of ML techniques in optimizing material properties, which can be directly translated to the research of SNCs for creating customized, high-performance functional foods.

Predictive analytics powered by ML also empower manufacturers to anticipate consumer needs and adjust production strategies in real time. For instance, purchase prediction models can identify specific consumer groups for targeted marketing, boosting both engagement and conversion rates. When applied to SNC-based functional foods, this approach ensures that products not only meet nutritional requirements but also resonate with consumer preferences for health and convenience [111].

## 6. Future Perspectives and Research Directions

**Figure 5:**
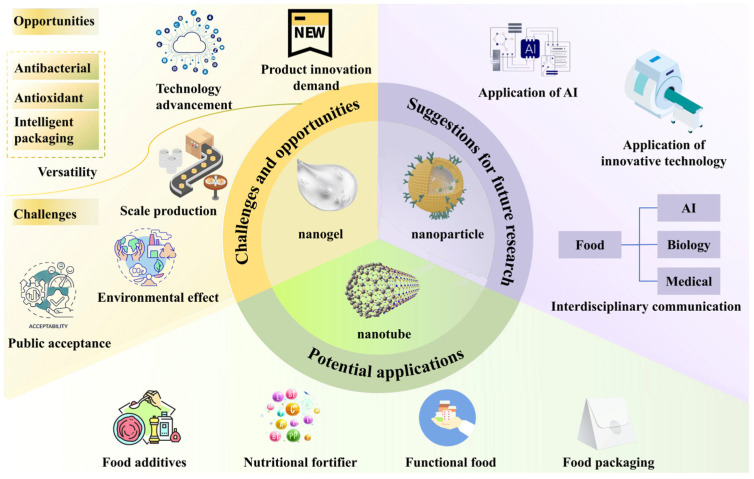
Opportunities, challenges, and future directions of starch-based nanocomposites in food science.

The future of SNCs lies in their potential to revolutionize functional food systems by enhancing stability, bioactivity, and sustainability. AI and NMR technology will continue to play a critical role in elucidating structural characteristics, monitoring molecular dynamics, and evaluating the interactions of bioactive compounds. However, to unlock the full potential of SNCs, overcoming commercialization challenges and advancing interdisciplinary research are imperative. Figure 5 summarizes the challenges and opportunities, potential applications, and suggestions for future research for SNCs, offering a clear outlook for ongoing advancements.

### 6.1. Opportunities for SNCs Development

The rapid evolution of food science, driven by public demand for safer, healthier, and more sustainable products, positions SNCs as a key innovation in the field. These materials bridge the gap between cutting-edge nanotechnology and practical food applications, offering solutions such as nutrient delivery systems, sustainable packaging, and low-calorie fat substitutes. The versatility of SNCs has fostered their integration into multiple disciplines [3,72], enabling innovations in food processing and packaging to extend shelf life and enhance product quality. Emerging technologies such as AI and advanced NMR techniques provide exciting opportunities for SNCs development. For instance, AI can optimize SNCs formulation processes through predictive modeling, ensuring precision and efficiency at every stage. By leveraging these technologies, the food industry can accelerate the adoption of SNCs and unlock their full potential in addressing health and sustainability challenges.

### 6.2. Challenges in Commercializing SNCs

#### 6.2.1. Scale Production

Despite the potential of SNCs, scaling up production while ensuring quality and cost-effectiveness remains a significant challenge. Researchers are exploring methods such as extrusion technology for cost-effective and efficient large-scale production of SNCs, which has shown promise in food applications [25]. Additionally, ultrasonic treatment has been explored to improve the dispersibility and stability of SNCs in food emulsions. For example, Guida et al. [112] demonstrated that ultrasonic treatment enhanced the stability of cassava starch nanoparticles, offering a more effective and scalable approach to their application in the food industry.

#### 6.2.2. Regulatory Landscape

Building consumer trust and ensuring regulatory compliance are critical to the widespread adoption of SNC-based products. Regulatory bodies, such as the European Food Safety Authority (EFSA) [113] and Food Standards Australia New Zealand [114], have established guidelines to ensure the safety of nanocomposites in food. Similarly, agencies like the Food and Drug Administration (FDA) [115], as well as regulatory authorities in China and Japan [116], have enacted corresponding laws and regulations to govern the use of nanocomposites in the food industry, such as requiring a comprehensive risk assessment of nanocomposites to ensure their safety. These efforts aim to reassure consumers and promote public confidence in the safety of SNCs. However, consumer trust is also influenced by cultural, legal, and informational factors, including the clarity of labeling. Studies by Parrella [117] emphasized that the effectiveness of transparency in labeling was a key factor in increasing consumer acceptance of nanotechnology in food products. The global regulatory landscape remains fragmented, with varying approaches to the safety and regulation of nanocomposites, which creates uncertainty for producers [114]. The United States Food and Drug Administration (FDA) [115] and other institutions, as well as the regulatory agencies in China and Japan [116], have formulated corresponding laws and regulations to manage the use of nanocomposites in the food industry, such as requiring a comprehensive risk assessment of nanomaterials to ensure their safety.

#### 6.2.3. Environmental Impact

To align with sustainability goals, it is crucial to develop environmentally friendly production methods for SNCs. Traditional production methods, such as acid hydrolysis [118] and emulsion cross-linking [119], are often energy-intensive and generate toxic by-products. Researchers are investigating green synthesis methods, such as ultrasonic-assisted synthesis and green chemistry techniques, to reduce environmental impact. Zhu et al. [120] used ultrasonic-assisted acetic acid hydrolysis to prepare starch nanocrystals, resulting in higher crystallinity and improved emulsion stability. Similarly, Khan et al. [121] synthesized starch-coated nanoparticles using plant extracts, avoiding chemical reducing agents and organic solvents.

#### 6.2.4. Comparative Analysis with Alternative Nanocomposites

When compared to alternative nanocomposites like cellulose, chitosan, and alginate-based nanoparticles, SNCs offer a more cost-effective and scalable solution due to their low-cost raw material, starch. However, they still face challenges related to processing complexity and consumer acceptance, especially when compared to well-established nanomaterials like cellulose and chitosan. While these materials have excellent mechanical properties and biodegradability, they are more expensive to produce, whereas SNCs offer a sustainable alternative with the potential for large-scale production.

### 6.3. Future Research Directions

#### 6.3.1. Interdisciplinary Communication

To fully realize the potential of SNCs, interdisciplinary collaboration between academia, industry, and regulatory bodies is essential. Collaborative efforts, such as those between companies and universities in agricultural research [122], have demonstrated the value of combining expertise from diverse fields to address global challenges. Similarly, partnerships between universities and medical institutions are paving the way for integrating food science into healthcare systems to improve public health outcomes [80].

#### 6.3.2. Application of AI and NMR

AI and NMR technologies have great potential in optimizing SNCs for food applications. Companies are already using AI to expedite product development, while Foodpairing utilizes AI to innovate new food formulations [123]. Additionally, NMR spectroscopy plays a key role in monitoring the stability of food ingredients during production, as demonstrated by collaborations with companies [124].

## 7. Conclusions

SNCs have emerged as a transformative advancement in food science, offering substantial improvements in stability, bioactivity, and functional properties within food systems. The integration of cutting-edge technologies, such as NMR and AI, has revolutionized the research and development of SNCs, enabling precise material design and functional optimization. NMR has proven invaluable in revealing the intricate structural and molecular dynamics of SNCs, while AI has introduced a powerful analytical framework that aids in predicting the stability, bioavailability, and health benefits of nanocomposites. This offers researchers the ability to design customized, high-performance nanocomposites that align with both health-driven requirements and market demands.

The continued development of SNCs is poised to address critical challenges in food science, such as enhancing nutritional content, improving food quality, and fostering environmental sustainability. Looking forward, the interdisciplinary collaboration between academia, industry, and regulatory bodies will be key to advancing the commercialization of SNCs. As these efforts progress, SNCs will undoubtedly play a pivotal role in redesigning functional food systems, providing consumers with healthier, safer, and more nutritious food options that also contribute to sustainable food production.

## Figures and Tables

**Table 2:** The applications of SNCs in foods.

Application	Compounds	Advantages	References
Delivery	Corn starch/lactobacillus rhamnosus	Enhancement of stability and vitality of probiotics.	[82]
	Corn starch/β-cyclodextrin/tea polyphenols	Improvement of stability and bioavailability of tea polyphenols.	[83]
	Amphiphilic starch synthesized by stearic acid and lauric acid	Stability improvement of hydrophobic molecules, and bioavailability enhancement in gastrointestinal tract.	[84]
	Chitosan/starch	A potential candidate for drug release control and fluorescence sensing applications.	[85]
	Zein/carboxymethyl starch/quercetin	Improvement of stability, bioavailability, and targeted delivery ability of quercetin.	[86]
	Carboxymethylation resistant starch/chitosan	Accomplishment of in situ gelation in stomach, biocompatibility, degradation resistance, and sustained drug release.	[87]
	Octenyl succinic anhydride-modified starch/curcumin	Remarkable improvement in solubility, stability, and bioavailability of curcumin.	[88]
	Amphiphilic hydroxyethyl starch/linoleic acid-modified berberine	Improvement in anti-cancer activity of modified berberine, and excellent ability to inhibit the expression of oncogene in zebrafish model.	[89]
Packaging	Starch/chitosan/taro mucus embedded with zinc oxide nanoparticles	Excellent mechanical properties, antibacterial activity, and biocompatibility.	[90]
	Films based on hydroxypropyl starch/polyvinyl alcohol loaded with zinc oxide nanoparticles	Excellent antibacterial properties, mechanical stability, and biodegradability.	[91]
	Corn starch/curcumin-loaded nanocomplexes	Improvement in postharvest quality and shelf life of blueberries.	[92]
	Konjac glucomannan/xanthan gum/soy protein isolate/tannic acid/iron	Excellent mechanical properties, barrier properties, and antibacterial and antioxidant activities.	[93]
	Corn starch/chitosan/cellulose nanofibers/cinnamon essential oil	Excellent mechanical, physical, and chemical properties, and significantly reduced bacterial load in beef packed with this film.	[94]
	Maize starch/polyvinyl alcohol/nano-clay/essential oil	Excellent antibacterial performance and oxidation resistance, degradable and environmentally friendly.	[95]
	Potato starch/modified banana fibers	Excellent mechanical properties, thermal stability, non-toxicity, and biodegradability, and effective extension of grape shelf life.	[96]
	Potato starch/carrageenan/co-pigment/anthocyanin	High stability, pH sensitivity, excellent optical, mechanical, and barrier properties, and remarkable antioxidant and antibacterial abilities.	[97]

## Data Availability

No new data were created or analyzed in this study. Data sharing is not applicable to this article.
